# Comparison of Smartphone and internet addiction and Optical Coherence Tomography findings among University students

**DOI:** 10.1192/j.eurpsy.2024.400

**Published:** 2024-08-27

**Authors:** Y. S. Atan, P. Güzel Özdemir

**Affiliations:** ^1^Psychiatry; ^2^Yüzüncü Yil Univercity, Van, Türkiye

## Abstract

**Introduction:**

Internet and smartphone use that reaches the level of addiction often leads to deterioration in the quality of life and functionality of individuals.

**Objectives:**

In our study, we aimed to investigate possible differences in retinal nerve fiber layer (RNFL) thickness and central macular thickness obtained by optical coherence tomography in internet and smartphone addiction.

**Methods:**

A total of 212 volunteer university students participated in our study. All participants were administered the Sociodemographic Information Form, Chen Internet Addiction Scale, Smartphone Addiction Scale-Short Form. Participants who completed the scales underwent routine eye examinations by experienced physicians in the ophthalmology outpatient clinic. Retinal nerve fiber layer (RNFL) thickness and central macular thickness were measured by optical coherence tomography (OCT).

**Results:**

In our study, internet addiction rate was 17% and smartphone addiction rate was 38.2%. RNFL thickness was found to be statistically significantly increased in the temporal superior and temporal inferior quadrants in those with internet addiction compared to healthy subjects (p<0.05). In smartphone addiction, RNFL thickness was found to be statistically significantly increased in the temporal inferior quadrant compared to healthy subjects (p<0.05). In the analyses comparing OCT measurements according to sex, it was found that nasal inferior (p<0.01) and global (p<0.05) quadrants in women and central macular thickness (p<0.01) in men were statistically significantly increased.

The correlation analyses in our study revealed statistically significant positive correlations between internet addiction scale scores (p<0.01) and smartphone addiction scale scores (p<0.01), RNFL temporal superior quadrant thickness (p<0.01); smartphone addiction scale scores and RNFL temporal superior quadrant thickness (p<0.05).

**Conclusions:**

Internet and smartphone addiction are seen considerable rates among university students. In OCT measurements, RNFL thickness was found to be increased in various quadrants in patients with addiction. In addition, RNFL thickness was found to be increased in all quadrants in female gender and central macular thickness was found to be increased in male gender. Correlation analysis revealed that internet addiction scale scores, smartphone addiction scale scores, and RNFL temporal superior quadrant thickness were positively correlated. In addition, there was a positive correlation between smartphone addiction scale scores, and RNFL temporal superior quadrant thickness

**Disclosure of Interest:**

None Declared

